# Habitat degradation alters trophic pathways but not food chain length on shallow Caribbean coral reefs

**DOI:** 10.1038/s41598-018-22463-x

**Published:** 2018-03-07

**Authors:** Piedad S. Morillo-Velarde, Patricia Briones-Fourzán, Lorenzo Álvarez-Filip, Sergio Aguíñiga-García, Alberto Sánchez-González, Enrique Lozano-Álvarez

**Affiliations:** 1 Unidad Académica de Sistemas Arrecifales, Instituto de Ciencias del Mar y Limnología, Universidad Nacional Autónoma de México, Puerto Morelos, Quintana Roo Mexico; 2 Centro Interdisciplinario de Ciencias Marinas, Instituto Politécnico Nacional, La Paz, Baja California Sur Mexico; 3 Present Address: CONACYT–Instituto de Ciencias Marinas y Pesquerías, Universidad Veracruzana, Boca del Río, Veracruz, Mexico

## Abstract

Habitat degradation can affect trophic ecology by differentially affecting specialist and generalist species, and the number and type of interspecific relationships. However, the effects of habitat degradation on the trophic ecology of coral reefs have received limited attention. We compared the trophic structure and food chain length between two shallow Caribbean coral reefs similar in size and close to each other: one dominated by live coral and the other by macroalgae (i.e., degraded). We subjected samples of basal carbon sources (particulate organic matter and algae) and the same 48 species of consumers (invertebrates and fishes) from both reefs to stable isotope analyses, and determined the trophic position of consumers and relative importance of various carbon sources for herbivores, omnivores, and carnivores. We found that both reefs had similar food chain length and trophic structure, but different trophic pathways. On the coral-dominated reef, turf algae and epiphytes were the most important carbon source for all consumer categories, whereas on the degraded reef, particulate organic matter was a major carbon source for carnivores. Our results suggest that the trophic structure of the communities associated with these reefs is robust enough to adjust to conditions of degradation.

## Introduction

One of most evident effects of habitat loss and degradation in terrestrial and aquatic ecosystems is a decline in the diversity of ecological communities via changes on species abundance and richness^1,2^. However, habitat degradation can modify the number of species interactions, potentially altering the trophic ecology^3,4^. For example, in a forest subjected to selective logging in Borneo, species of ground-feeding and understorey-feeding birds had significantly higher trophic positions than they had in a non-logged forest^5^. In Moorea, the stable isotopic signatures of marine carbon sources and consumers differed significantly between two bays as a result of different levels of anthropogenic activities causing differences in mean annual river flow to each bay^6^.

Life history traits may also determine the response of species to habitat degradation, with specialist species generally being more affected than generalist species^7,8^. For example, butterfly species with a narrow feeding niche and low levels of mobility and reproduction were most strongly affected by habitat loss across a wide range of habitats in America and Europe^9^. Also, among bird species, long-lived, large, non-migratory, forest specialists were less likely to occur and less abundant in more intensively man-used habitats than were short-lived, small, migratory, habitat generalists^10^.

Coral reefs are the most biologically diverse ecosystems in tropical waters and provide important ecosystem services to millions of people around the world^11^. However, coral reefs are being widely affected by a combination of global and local stressors, including climate change-induced coral bleaching, diseases, overfishing, and eutrophication^12^. Habitat degradation on coral reefs is mainly manifested as declines in the abundance of reef-building corals and their replacement by macroalgae or other organisms^13–15^. Coral reef degradation is already affecting community structure by changing diversity and abundance of species^16,17^ as well as ecosystem functioning and services^18,19^. The removal of particular species (e.g., by overfishing) and the addition or increase in abundance of others may fundamentally change ecological feedbacks, resulting in a transition of the ecosystem to an alternate state^4,20^. As occurs with terrestrial species, coral reef specialists are expected to be more affected than generalists by reef degradation^21–23^, further altering the food webs^16^.

Food-chain length is an important descriptor of community structure and ecosystem functioning^24,25^. Ecosystem size and disturbance have been examined as factors potentially determining food chain length in some aquatic and terrestrial ecosystems, with results pointing to ecosystem size as having a greater influence than disturbance^26,27^. Yet, at the global scale, food chain length showed weak or no relationships with ecosystem size^28^, probably because environmental variables interact in complex ways to structure a community and may affect metrics of food web structure other than food chain length^29^.

Stable isotopes provide information on the flow of energy or nutrients through food webs^30^ and a measure of food-chain length that integrates the assimilation of energy or mass flow through all the trophic pathways leading to top predators (i.e., the trophic structure)^25,26^. Carbon isotope ratios are used as a tracer of food carbon source, whereas nitrogen isotope ratios are indicative of consumer trophic position^31^. The use of stable isotope analyses and descriptors (e.g., trophic niche size) to examine the trophic structure and functioning of coral reef organisms and communities has been increasing in the last few years^32–34^. For example, some studies have examined the stable isotope composition of reef organic matter sources and consumers along environmental gradients or in different seasons^35–38^. Others have examined the potential effect of habitat degradation on coral reef food webs via stable isotope analyses by focusing on species of higher order consumers^34^.

**Figure 1:**
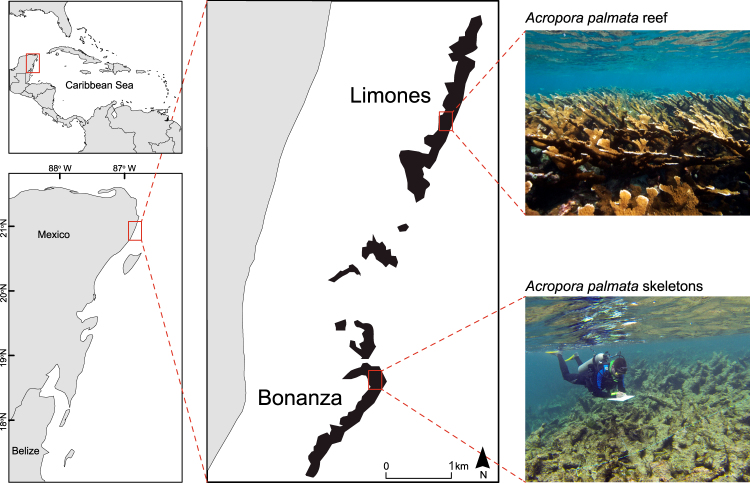
General location and study sites. (**upper left panel**) Caribbean region, (**bottom left panel**) Mesoamerican Reef, (**central and right panels**), Limones and Bonanza reefs on the Caribbean coast of Mexico. Map produced in QGIS 2.18 (www.qgis.org) using the following data sources: National Geospatial-Intelligence Agency (base map, World Vector Shoreline Plus, 2004. http://shoreline.noaa.gov/data/datasheets/wvs.html). The location of survey sites was obtained from the present study. Data sources are open access under the Creative Commons License (CC BY 4.0).

Here, we analyse the effect of habitat degradation on food webs by comparing the trophic structure and food chain length between two shallow Caribbean coral reefs known as “Limones” and “Bonanza” that are similar in size and subjected to the same environmental conditions, but have contrasting levels of degradation (Fig. 1). Limones is known for its abundance of the reef-building coral *Acropora palmata*^39^. Contrarily, Bonanza, which previously held abundant colonies of *A. palmata*, has sustained a substantial decline in live coral cover and increase in macroalgal cover from values estimated in 1985 (33% and 4%, respectively)^40^. We first compared the architectural complexity (rugosity index) and percent cover of live coral and different types of functional groups of algae between reefs. We then explored whether the trophic structure and food chain length of associated reef communities differed between Limones and Bonanza by comparing the stable isotopes (δ^15^N and δ^13^C) of several basal carbon sources and the same 48 reef-associated consumer species on both reefs as well as the trophic position of the latter. Using bi-plots of δ^13^C and δ^15^N values we compared the isotopic niche widths of the different trophic categories of consumers (herbivores, omnivores, carnivores) between reefs by means of several metrics of trophic structure^41,42^. We hypothesized that, as the coral-dominated Limones provides more habitats and trophic niches than Bonanza, then it will also exhibit a more complex trophic web with a higher trophic position of predators than the degraded reef.

## Results

### Characterization of reef and benthic composition

**Figure 2:**
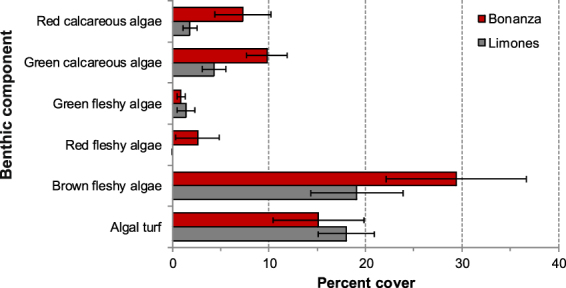
Macroalgal abundance on each reef. Percent cover of different functional groups of macroalgae on Limones (grey columns) and Bonanza (red columns) reefs (N = 8 transects per reef; error bars denote 95% confidence intervals).

As anticipated, the ecological condition of both reefs differed broadly (Fig. 1). Live coral cover was significantly higher on Limones (50.1 ± 15.1%, mean ± 95% CI) than on Bonanza (7.3 ± 4.6%) (t_14_ = 5.46, p = 0.0003), as was reef rugosity (Limones: 1.92 ± 0.31; Bonanza: 1.42 ± 0.09; t_14_ = 3.0, p = 0.025); whereas cover of brown and red fleshy, and green and red calcareous macroalgae was significantly greater on Bonanza than on Limones (Fig. 2). The average cover of all macroalgae (defined as erect fleshy or calcareous algae larger than 2 cm, i.e., excepting algal turf^15^) was greater on Bonanza (69.9%) than on Limones (26.6%).

### Trophic structure and food chain length

**Figure 3:**
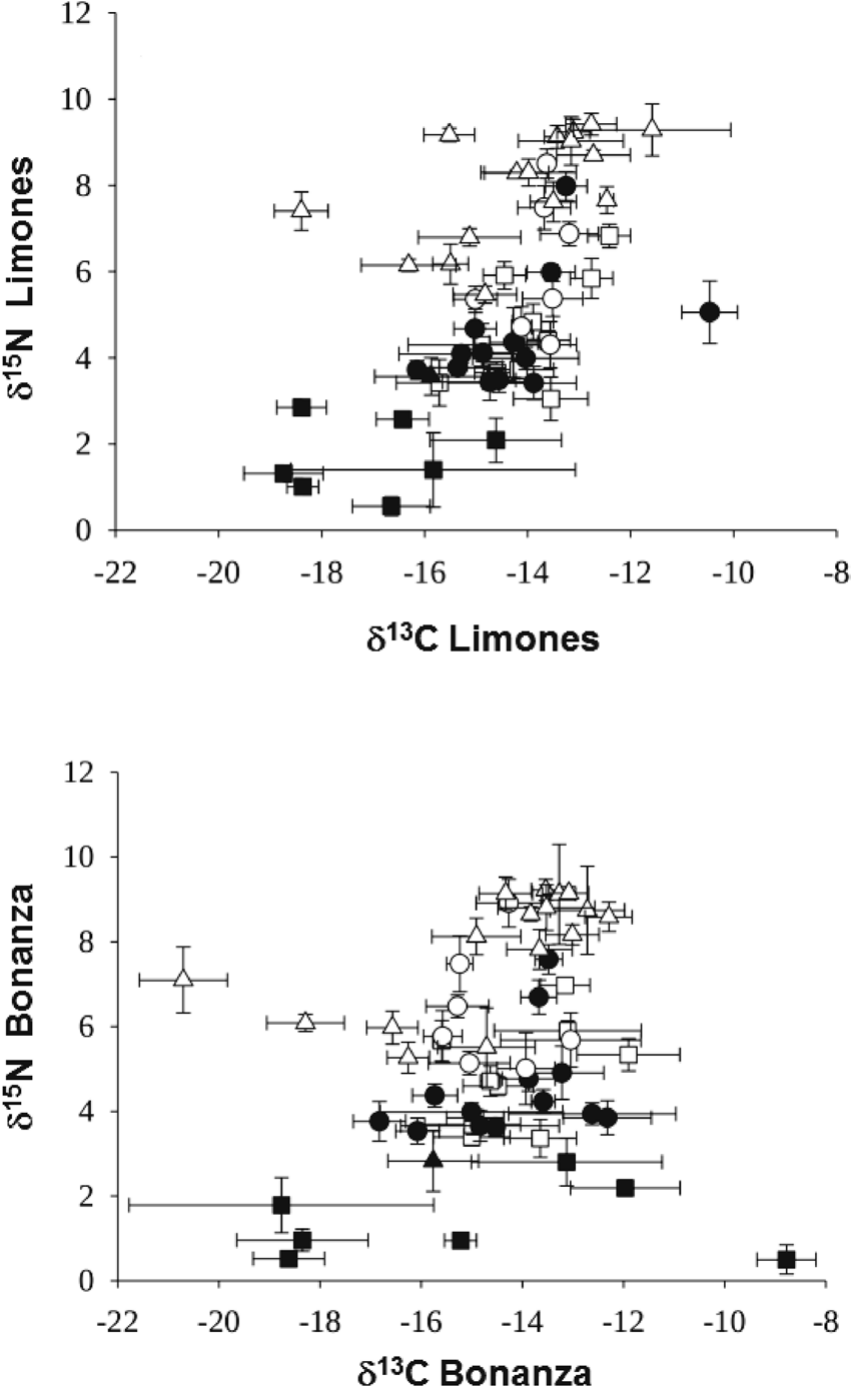
Trophic space for studied reefs. Biplot of mean ± SD δ^13^C and δ^15^N values for basal carbon sources (filled squares), annelids (filled triangles), crustaceans (open squares), mollusks (filled circles), echinoderms (open circles), and fishes (open triangles) on Limones (upper panel) and Bonanza (lower panel) reefs.

**Figure 4:**
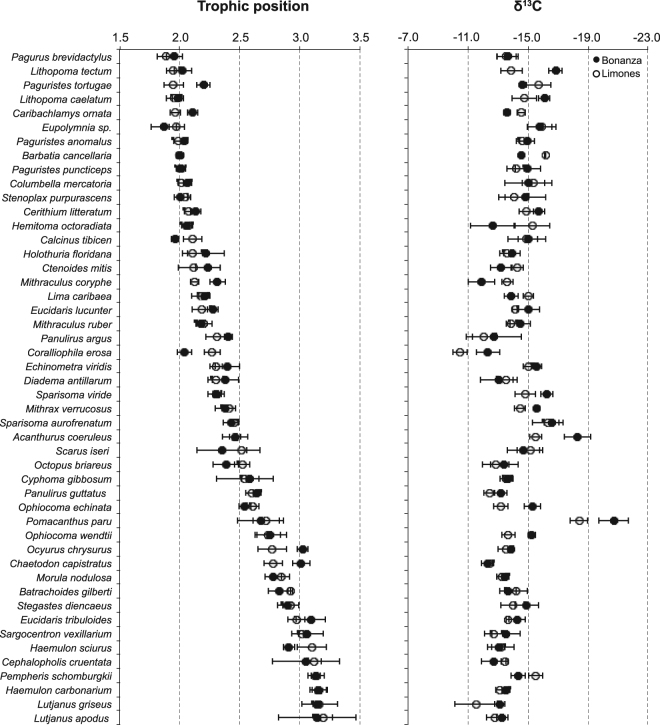
Food wed model for Limones and Bonanza reefs. Mean ± 95% confidence interval for trophic position (left panel) and δ^13^C values (right panel) of 48 species of consumers on Limones (open circles) and Bonanza (filled circles) reefs.

Despite the differences in cover of benthic components, the stable isotope analyses showed that the trophic structure of Limones and Bonanza was generally similar (Fig. 3). However, whereas the overall range of δ^15^N values was very similar on both reefs, the range in δ^13^C values was wider on Bonanza than on Limones (Fig. 3, Supplementary Table S1). On both reefs, basal carbon sources had a similar range of δ^15^N values (0.52 to 2.85‰) (Supplementary Table S1). Among consumers, the lowest δ^15^N values were obtained for herbivorous and omnivorous invertebrates such as the terebellid polychaete *Eupolymnia* sp., the hermit crab *Pagurus brevidactylus*, and the star snails *Lithopoma tectum* and *L. caelatum*, whereas the highest δ^15^N values were obtained for four carnivorous fishes: the schoolmaster snapper *Lutjanus apodus*, the grey snapper *L. griseus*, the Caesar grunt *Haemulon carbonarium*, and the glassy sweeper *Pempheris schomburgkii* (Supplementary Table S1). Estimates of trophic positions (TP) generally matched the dietary information of these species reported in the literature (Supplementary Table S1). In only eight of the 48 consumer species the TP varied significantly with reef. Five of these species had a higher TP on Bonanza (the scallop *Caribachlamys ornata*, the hermit crab *Paguristes tortugae*, the nodose clinging crab *Mithraculus coryphe*, the yellow-tail snapper *Ocyurus chrysurus*, and the four-eye butterflyfish *Chaetodon capistratus*), whereas three had a higher TP on Limones (the orangeclaw hermit *Calcinus tibicen*, the muricid snail *Coralliophila erosa*, and the bluestriped grunt *Haemulon sciurus*). Regardless, there was a high correlation between the TP values of individual consumers from Limones and Bonanza (*r* = 0.97) (Fig. 4). Moreover, the TP values of the five species of carnivores with the highest TP did not differ significantly between reefs (Fig. 4, Supplementary Table S1), indicating a similar food chain length.

The overall range in δ^13^C values on the degraded reef (Bonanza) relative to Limones reflects the broader range in δ^13^C values of basal carbon sources, which ranged from −18.76 ± 3.01 (mean ± SD) for turf algae to −8.78 ± 0.58 for the red coralline algae *Amphiroa tribulus* (Supplementary Table S1). The δ^13^C values of carbon sources were generally more enriched on Bonanza, except for algal turf, the green fleshy alga *Caulerpa racemosa*, and the green calcareous alga *Halimeda tuna* (Supplementary Table S2). On both reefs, the more depleted δ^13^C values among consumers were those for the French angelfish *Pomacanthus paru*, the blue tang *Acanthurus coeruleus*, the star snail *L. tectum*, and the redband parrotfish *Sparisoma aurofrenatum*, and the more enriched for *C. erosa* and *M. coryphe* (Supplementary Table S1). Significantly more enriched δ^13^C values were found for 10 species (nine invertebrates and one fish) on Limones and for eight species (seven invertebrates and one fish) on Bonanza (Supplementary Table S2). Therefore, the correlation between the δ^13^C values of consumers between both reefs was lower (*r* = 0.69) than the correlation between the corresponding TP values (Fig. 4).

### Importance of basal carbon sources to consumers

**Figure 5:**
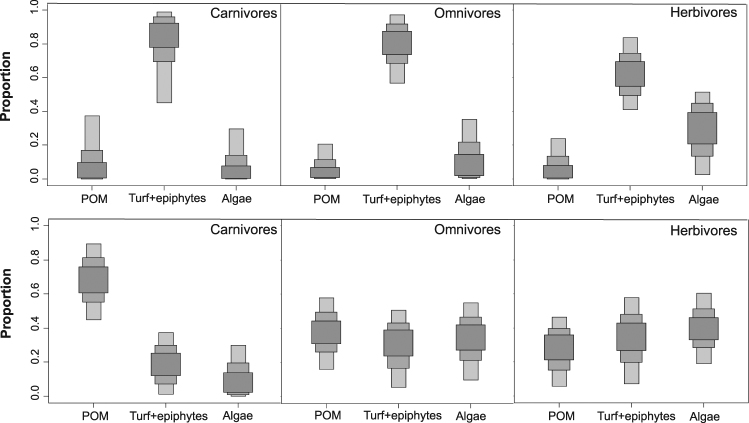
Importance of carbon sources for consumers. The relative importance of basal carbon sources for carnivores, omnivores, and herbivores in Limones (upper panel) and Bonanza (lower panel) reefs. Boxes in different gray shading denote 95% (light), 75% (darker) and 50% (darkest) credibility intervals.

The relative importance of basal carbon sources supporting the food webs on each reef was explored with a Bayesian mixing model. This analysis revealed that the main carbon source for all three consumer categories on Limones was algal turf + epiphytes, which on average contributed approximately 60%, 80%, and 85% of organic carbon to the isotopic signature of herbivores, omnivores, and carnivores, respectively. Macroalgae were the second most important carbon source for herbivores (~29%), whereas POM contributed much smaller percentages to the isotopic signature of all consumer trophic categories on Limones (Fig. 5A). In contrast, at Bonanza all basal carbon sources contributed more evenly to the diet of omnivores and herbivores, but POM emerged as a more important source for carnivores, contributing ~70% to their isotopic signature versus ~20% from turf + epiphytes and ~10% from macroalgae (Fig. 5B).

### Trophic niche of consumers

**Figure 6:**
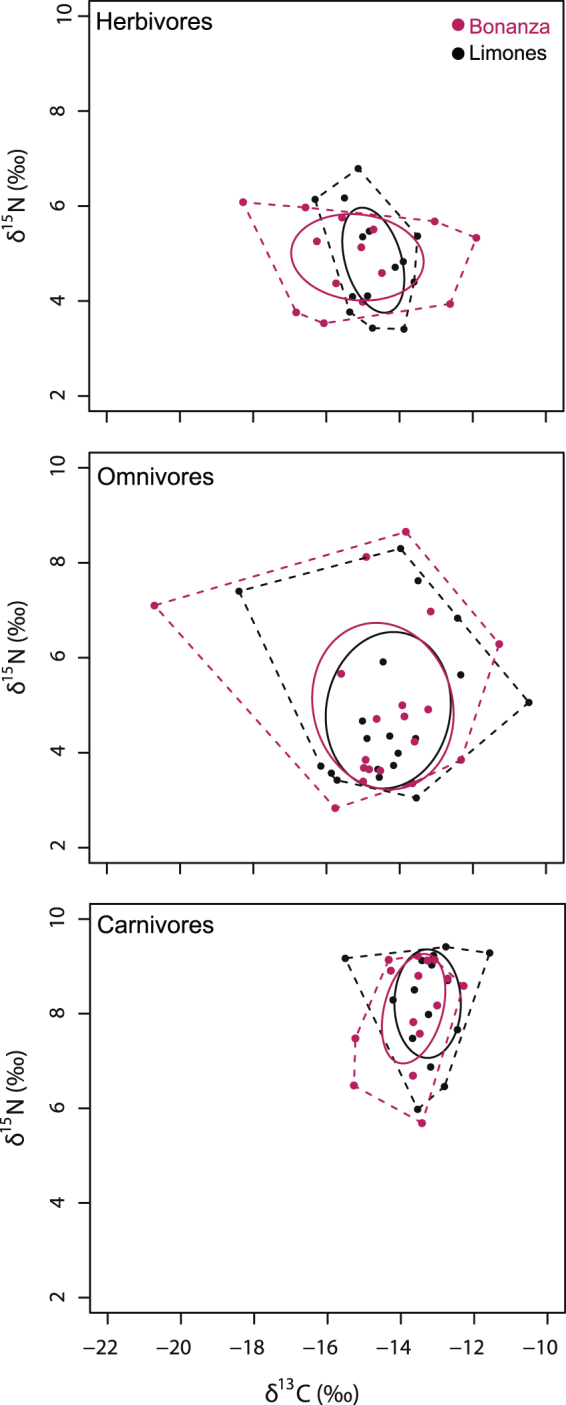
Isotopic niche by trophic category of consumers. Mean δ^13^C and δ^15^N values of herbivores (top panel), omnivores (center panel) and carnivores (bottom panel) in Limones (black) and Bonanza (red) reefs. Dotted lines: convex hull area (TA). Solid lines: standard ellipse area corrected for small sample sizes (SEAc).

The total area of the trophic niche for each consumer category was compared between reefs. The difference was much greater for herbivores than for omnivores and carnivores (Fig. 6). Herbivores from Limones had a relatively narrow δ^13^C range (from −16.31‰ for *S. aurofrenatum* to −13.51‰ for the long-spined sea urchin *Diadema antillarum*) and a smaller standard ellipse area (SEAc) (Table 1). In contrast, herbivores from Bonanza had a broader δ^13^C range (from −18.28‰ for *A. coeruleus* to −11.90‰ for *M. coryphe*), resulting in a greater niche size, as indicated by the convex hull area (TA) and SEAc (Table 1), with much less overlap (47%) between their SEAc and the SEAc of herbivores from Limones than the other way around (88%) (Table 1, Fig. 6). In omnivores, the magnitude of the TAs was large because *P. paru* was relatively depleted in δ^13^C on both reefs, but more so in Bonanza, constituting an outlier and resulting in a relatively large convex hull in which much of the contained niche space was unoccupied. In this case, the SEAc provides a better characterization of the niche width^30^. The SEAc of omnivores from Limones and Bonanza had a great overlap (100% and 84%, respectively) (Table 1, Fig. 6). TAs were much smaller for carnivores than omnivores, and SEAc of carnivores showed a substantial overlap between reefs (69% for Limones and 76% for Bonanza) (Table 1, Fig. 6). Overall, these results strongly suggest that Bonanza has a greater range of benthic basal carbon sources than Limones, which is consistent with the differential percent cover of macroalgae between reefs (see Fig. 2).

**Table 1:** Isotopic niche metrics for consumers on Limones and Bonanza reefs. NR (δ15N range), CR (δ13C range), convex hull total area (TA), Bayesian standard ellipse area (SEA), Bayesian-corrected estimate of the standard ellipse area (SEAc), overlap in SEAc between reefs for each category, and percentage of overlap with SEAc of the same trophic category from the other reef.

Trophic category	Reef	NR	CR	TA units²	SEA units²	SEAc units²	SEAc overlap units² (%)
Herbivores	Limones	4.88	2.80	6.06	2.54	2.75	2.42 (88%)
	Bonanza	4.59	6.58	11.00	4.75	5.15	2.42 (47%)
Omnivores	Limones	6.12	6.31	24.56	8.33	8.82	8.78 (100%)
	Bonanza	6.01	7.55	32.02	10.01	10.60	8.78 (84%)
Carnivores	Limones	4.36	3.75	7.45	3.03	3.26	2.25 (69%)
	Bonanza	5.38	2.39	6.93	2.76	2.97	2.25 (76%)

## Discussion

Habitat degradation is thought to be a major driver of change in the species composition and trophic structure of ecosystems^2,5,9^. In coral reefs, recent studies have shown that alterations to habitat structure may result in the shortening of food chains through a decrease in the size of reef fishes^16^, eutrophication^33,43^, or changes in prey availability for mesopredators^34^. Here, we show that the food chain length and the trophic space of 48 reef-associated species were essentially unaltered when comparing a coral-dominated reef (Limones) and a degraded, macroalgal-dominated reef (Bonanza). We chose two reefs of a similar size to minimize the potential effect of ecosystem size on food chain length^24,27^. Our results also do not imply similarities in community structure because we intentionally sampled the same species on both reefs. Yet, the structure of the coral reef food web and the trophic position of high-order consumers (mesopredators) were similar between both reefs despite the difference in degradation, although consumers showed greater enrichment of δ^13^C on the degraded reef.

On coral reefs, baseline carbon sources may include benthic macroalgae, reef-associated detritus, particulate organic matter (POM), coral tissue, and phytoplankton^38,44^. We focused our samples of baseline carbon sources on erect macroalgae and turf, which cover a large percentage of reef area on both reefs (see Fig. 2), and on POM, which is an important carbon source in many coral reefs^45,46^. In a coral reef of New Caledonia, Le Bourg *et al*.^46^ compared food chains based on POM between two sites distant from each other by less than 10 km, a reef lagoon and the outer slope of the reef. They found that δ^15^N did not differ between zones, whereas δ^13^C values were significantly higher in the lagoon than on the outer slope, suggesting that the two food chains were based on different primary sources of carbon^46^. We obtained a similar result, as values of δ^15^N and the derived trophic positions of species did not differ between Limones and Bonanza but the contribution of different carbon sources varied substantially between the two food chains, even though we compared the same zone (back reef) of two separate reefs distant from each other by ~2 km.

Although results from mixing models should be taken with caution because they are based on fixed trophic enrichment factors^47^, our results strongly suggest that on the coral-dominated reef, turf algae and epiphytes constitute a key source of carbon for consumers of the different trophic categories, whereas macroalgae and POM are considerably less important. Turf is an important component of coral reef food webs because many more herbivorous fishes feed on turf algae than on erect macroalgae^48,49^, which they often find unpalatable. In addition, turf traps vast amounts of detritus, and some fishes that are considered as herbivorous actually feed on the detritus trapped in the turf^50^. Also importantly, algal turf constitutes one of the most productive habitats for small reef mesograzer invertebrates, in particular small crustaceans such as amphipods, isopods, ostracods, and tanaids^51,52^, which can be consumed by fishes^53,54^. Therefore, turf-feeding herbivores constitute an important trophic link between benthic primary production and secondary consumers^55,56^, especially since the use of stable isotopes has revealed that predators on coral reefs consume more herbivorous prey (both fishes and invertebrates) than previously reported^57^. This appears to be the case in Limones, where the isotopic signature of consumers in all trophic categories was consistent with a more benthic-derived carbon pathway. Similarly, benthic primary production was found to be an important source for consumer production, including apex predators, in some coral-dominated coral reefs of Australia and Hawaii^32,35,38^.

In contrast to Limones, the distribution of the carbon signal on Bonanza was broader and originated from different sources, potentially increasing trophic diversity (indicated by a broader range in δ^13^C) at the base of the food web^41^. Among consumers, herbivores in particular exhibited a broader isotopic niche along the carbon axis on Bonanza compared with Limones. On Bonanza, macroalgae, algal turf, and POM were identified as being more or less equally important as carbon sources for herbivores and omnivores, but not for carnivores. Degraded reefs are characterized by increases in algal cover that can benefit some herbivorous fishes but only in the short term, as some large erect macroalgae (such as species in the genera *Dictyota*, *Lobophora*, *Laurencia*, and *Sargassum*) are often unpalatable to fishes^58,59^. These macroalgae are readily consumed by small mesograzers and omnivores, such as sea hares, some snails, amphipods, and majoid crabs^60,61^, but the ecological effects of these small animals tend to be more localized than those of herbivorous fishes or urchins^62^. Therefore, macroalgal-feeding herbivores may not represent a major food source for carnivores on Bonanza, for which POM was identified as the main carbon source. On coral reefs, POM includes phytoplankton as well as other edible particles such as phytobenthos debris, mucus, and faeces^45^. Small pelagic and benthic fishes associated with coral reefs, such as silversides (Atherinidae) and basslets (Grammatidae), feed on POM and zooplankton, as do juveniles of surgeonfishes (Acanthuridae), grunts (Haemulidae), jacks (Carangidae), and yellowtail snappers (*Ocyurus chrysurus*)^63^. Other fishes that are mainly herbivorous can change feeding modes depending on the ecological context^58,64^. These fishes are prey for many reef mesopredators, which may further use resources differentially depending on the seascape configuration. For instance, the blackspot snapper *Lutjanus ehrenbergii* shifted from a benthic macroalgal food web on shelf reefs to a phytoplankton-based food web on oceanic reefs in the Red Sea^44^. Planktonic-derived production was also identified as important for sustaining key predatory species in reef fisheries in Australia^45,65^. On Bonanza, reef degradation appears to be causing a temporal shift from a benthic algal-based food web to a phytoplankton-based food web.

Omnivory is common in marine food webs^66^ and many of the species that we sampled on both reefs were omnivores with broader isotopic niches than those of herbivores or carnivores, as would be expected. On both reefs, the wider niche breadth of omnivores was mainly caused by the extreme position in the niche space of the carbon-depleted French angelfish, *P. paru*. Marine benthic consumers that rely upon phytoplankton for sustenance will generally display low (depleted) δ^13^C values^67^. French angelfish can consume algae and invertebrates, but feed mostly on sponges^63^, which are filter feeders and hence display low values of δ^13^C. For example, an average δ^13^C value of −17.2‰ was recorded for coral reef sponges in Florida^68^. Nevertheless, even after accounting for outliers such as *P. paru*, omnivores from Bonanza had the largest overall isotopic niche as revealed by the size of the SEAc. Omnivores play an important role in dampening potential trophic cascades^69^ and would be expected to have a greater potential of short-term adaptive response to changes in habitat degradation.

Phase shifts are becoming increasingly common in coral reefs with different levels of habitat degradation^70,71^. The current paradigm is that habitat loss and degradation are the main drivers of biodiversity loss, but our results cannot be discussed in terms of diversity or abundance because that was not the aim of our study. We found that coral cover was much lower and macroalgal cover much higher in Bonanza than in Limones. Yet, despite the differences in habitat degradation, food chain length was similar on both reefs, suggesting that the trophic structure of the communities associated with these reefs is robust enough to adjust to conditions of degradation. However, we do not interpret our finding as indicative of a lack of effect of habitat degradation on the trophic ecology of these reefs, because the range in δ^13^C values for carbon sources and herbivorous species was wider in Bonanza than in Limones, and the carbon pathways appear to differ between both reefs despite their proximity. Future research should include sampling on more coral- and algal-dominated reefs to examine the generality of our findings, and exploring the presence of potential sublethal effects (e.g. lower nutritional condition^59,72^) of habitat degradation on high-order consumers.

## Methods

### Study site

The study was conducted in the Puerto Morelos Reef National Park, located on the Mexican portion of the Mesoamerican Reef System (see Fig. 1). The Puerto Morelos reef system encompasses a series of shallow reef units and patches constituting an extended fringing reef system separated from the shoreline by a shallow lagoon. There is more coral cover on the crest and back-reef zones (down to approximately 5 m in depth) than on the fore-reef zone, which is mostly of low relief^40^. Habitat degradation varies among reef units and patches^23^. We selected two reef units exhibiting visibly contrasting levels of degradation: “Limones” and “Bonanza”. Limones (centred at 20°59.1′N, 86°47.9′W) is considered an exceptional site for *Acropora palmata* within the Mesoamerican Reef System due to its large populations of this branching coral species that cover nearly 40% of the reef substrata^39^ (Fig. 1). In contrast, Bonanza (centred at 20°57.6′N, 86°48.9′W), which in 1985 had 33% cover of live coral and 4% cover of algae^40^, currently exhibits extensive areas of dead *Acropora* skeletons (Fig. 1) and a predominance of erect macroalgae. Fishing activities are banned on both reefs since 1996. Given the high ecological value of Limones, tourist activities are not allowed in this reef since 2014^39^, whereas Bonanza is open to visitation. The southern limit of Limones is separated from the northern limit of Bonanza by a distance of ~1900 m. The two reefs are similar in size (~1500 m in length), depth range (1–5 m), and distance from the shoreline. Samples for stable isotope analyses (water, plants, invertebrates, and fishes) were collected on the back reef to crest zones within an area of ~500 m^2^ near the centre of each reef.

### Characterisation of reef and benthic composition

To compare the current status of Limones and Bonanza, benthic habitat composition and architectural complexity were described for each reef using eight randomly selected 10-m transects within the same area in which samples were collected on each reef. At each transect, coral cover was measured by means of the line-point counts method. A surveyor recorded the benthic component (live coral in general and different functional groups of algae) intercepting the line every 10 cm (i.e., 100 points per transect), and cover of each component was estimated as a percentage of the number of points overlaying the transect. Also, at each transect, reef complexity was measured using the rugosity index, which is the ratio of a length of a chain moulded to the reef surface to the linear distance between its start and end point^18^. A perfectly flat surface would have a rugosity index of one, with larger numbers indicating more complex surfaces.

### Sample collection and preparation

The same species of consumers and types of basal carbon sources were sampled on both reefs by SCUBA diving and transported to the laboratory at UASA-UNAM (Puerto Morelos, Mexico) to be processed for stable isotope analyses. Consumers included 48 species, of which 32 were of invertebrates (including annelids (1 species), bivalves (4), gastropods (8), chitons (1), octopuses (1), decapod crustaceans (10), sea urchins (4), brittle stars (2), and sea cucumbers (1 species)) and 16 were of fishes (see Supplementary Table S1 for the full list of species). Our main criterion for selecting consumer species was that they were reef-associated species, preferentially with limited movement ranges, both to reduce the possibility of organisms shifting between reefs and because this type of species can be presumably more susceptible to reef habitat degradation. Basal carbon sources included particulate organic matter (POM), four species of macroalgae (*Amphiroa tribulus*, *Caulerpa racemosa*, *Dictyota cervicornis*, *Halimeda tuna*), epiphytes, and turf algae. For all consumer species and basal carbon sources, at least three to five replicates were sampled from each reef, yielding 514 samples in total. To minimize potential seasonal effects on the isotopic composition of organisms, all samples were obtained between late September 2015 and early February 2016, and samples from both reefs were interspersed throughout this period.

POM was obtained from seawater collected with a dark 5-L carboy. Then, 1 L of seawater was gently vacuumed using Whatman GF/F (0.7 µm) filters previously incinerated at 450 °C for 4 h. Samples of algal turf were taken from the top of dead coral using scalpel and forceps under a magnifying glass to avoid the presence of contaminants (e.g., endolithic animals)^73^. Macroalgae were collected by hand. Epiphytes were carefully removed from macroalgal fronds using a glass slide. Invertebrates were collected by hand or with the help of tweezers or nets, and transported live to the laboratory, where they were kept in containers with filtered seawater for 24–48 h with the aim of emptying their gut contents, which can affect the isotopic composition^74^. Individuals were then frozen and preserved at −20 °C. Prior to analyses, the calcareous shells or exoskeletons of invertebrates were removed. A sample of muscle was taken from the abdomen of crustaceans, the foot of chitons, gastropods and bivalves, the Aristotle’s lantern of urchins and brittle stars, the body wall of sea cucumbers, and the tentacles of octopuses. Fishes (16 species) were identified *in situ* and caught with Hawaiian spears. A sample of muscle was taken from the dorsal region and immediately frozen until processing^75^. All samples were acidified with HCl (1 N) to remove inorganic carbon^76^ and then rinsed with Milli-Q water. Samples were dried at 60 °C for 48 h in aluminium foil trays, then ground to a fine powder and homogenized with agate mortar and pestle. Powdered sub-samples were weighed and sent for isotopic analysis in ultra-pure tin capsules.

### Stable isotope analyses

Elemental and stable isotope analyses were carried out in a Finnigan Delta V Plus mass spectrometer (Thermo Scientific) interfaced with an elemental analyser (Elemental Combustion System, Costech model 4010) at the Mass Spectrometry Laboratory of Centro Interdisciplinario de Ciencias Marinas, Instituto Politécnico Nacional, La Paz, Mexico. The average precision across runs was 0.1‰ for δ^15^N and 0.02‰ for δ^13^C. Carbon and nitrogen ratios were expressed in delta (δ) notation, defined as parts per mil (‰) of difference relative to an international standard:

1 $${\rm{\delta }}X=\frac{1000({{\rm{R}}}_{{\rm{sample}}}-{{\rm{R}}}_{{\rm{std}}})}{{{\rm{R}}}_{{\rm{std}}}}$$

where X is N (nitrogen) or C (carbon), and R is the ratio of the heavier, rarer isotopes (^13^C and ^15^N) to the lighter, more common isotopes (^12^C and ^14^N, respectively). Delta values are reported relative to the international standards of Vienna Pee-Dee Belemnite (VPDB) carbon and atmospheric nitrogen^26^.

### Data analyses

Student’s *t* tests were used to test the null hypotheses of no significant differences in the mean percent coral cover, rugosity index, and δ^15^N and δ^13^C composition of each basal resource and consumer species between Limones and Bonanza. Bi-plots of δ^13^C and δ^15^N values of basal carbon sources and consumers were used to visualize the food web structure at each reef and the differences in isotope values of basal carbon sources and consumers among reefs.

The relative trophic position (TP) of all species of consumers from each reef was calculated using the following equation from the meta-analysis performed by Hussey *et al*.^77^:

2 $${\rm{TP}}=\frac{\mathrm{log}({\delta }^{15}{N}_{lim}-{\delta }^{15}{N}_{base})-\,\mathrm{log}({\delta }^{15}{N}_{lim}-{\delta }^{15}{N}_{TP})}{k}+{{\rm{TP}}}_{base}$$

where TP_*base*_ was set at a trophic level 2 baseline using the mean δ^15^N of the epifaunal reef clam *Barbatia domingensis* (=*B. cancellaria*), which we chose as the baseline organism given that filter-feeding bivalves are good integrators of the isotopic variation at the base of pelagic and benthic food webs^26,35^; δ^15^N_*lim*_ is the saturating isotope limit as TP increases; δ^15^N_*base*_ is the isotope value for a known baseline consumer in the food web (in this case, *B. domingensis*), and *k* is the rate at which δ^15^N_*TP*_ approaches δ^15^N_*lim*_ per TP step. Estimates of *k* and δ^15^N_*lim*_ are given by:

3 $$k=-\mathrm{log}(\frac{{\beta }_{0}-{\delta }^{15}{N}_{lim}}{-{\delta }^{15}{N}_{lim}})$$

4 $${{\rm{\delta }}}^{15}{N}_{lim}=\frac{-{\beta }_{0}}{{\beta }_{1}}$$

with values for the intercept β_0_ = 5.924 and the slope β_1_ = −0.271. These values characterize the change in δ^15^N as dietary δ^15^N values increase^77^.

To quantify the relative importance of basal carbon sources supporting the food webs on each reef, we applied a Bayesian mixing model using SIAR (Stable Isotope Analysis in R) version 3.0.2^78,79^. Mixing models require the use of trophic enrichment factors and for these purposes we used the mean (±SD) values proposed by Post^26^ for δ^13^C (0.40 ± 1.30‰) and δ^15^N (3.40 ± 1.00‰). The estimated values of the dietary proportion were obtained via a Markov-Chain Monte Carlo (MCMC) simulation^78^ on stable isotope data from consumers. Each consumer species was previously categorized as herbivore, omnivore, or carnivore based on literature reports on its type of diet (see Supplementary Table S1). For each trophic category on each reef, we calculated NR (δ^15^N range) and CR (δ^13^C range), measures that provide information on the food chain length and maximum trophic position within the community, and the diversity of basal carbon sources, respectively. We also inferred the total isotopic niche width using the total area (TA) index, which is a measure of the area of a polygon drawn through the most extreme data points of the population in the isotopic niche space (i.e., the convex hull)^41^, and the standard ellipse area (SEA), which measures the isotopic niche width of the mean core community and contains approximately 40% of the data^42^. To compare the total trophic niche area for each consumer trophic category (i.e., herbivores, omnivores, carnivores) between reefs, we used the Bayesian standard ellipse area corrected for sample size (SEAc), estimated and plotted using the SIBER routine for the SIAR package in R^42^. For each trophic category, distinction between the two SEAc for each reef was inferred by posterior probabilities derived by Bayesian inference based on 100,000 MCMC draws, and the trophic niche overlap was calculated as the proportion of SEAc overlapping between the two reefs.

### Data availability

All data generated or analysed during this study are included in this published article (and its Supplementary Information files).

## Electronic supplementary material

Supplementary information
