# Supplementary material for: Habitat degradation alters trophic pathways but not food chain length on shallow Caribbean coral reefs
Source: Sci Rep. 2018 Mar 7;8:4109. doi: 10.1038/s41598-018-22463-x (PMC5841442; doi:10.1038/s41598-018-22463-x)
Supplement: Supplementary file 1 — Supplementary information [file 41598_2018_22463_MOESM1_ESM.pdf]

Habitat degradation alters trophic pathways but not food chain length on shallow Caribbean coral reefs

Piedad S. Morillo-Velarde[a, c], Patricia Briones-Fourzán[a], Lorenzo Álvarez-Filip[a], Sergio Aguíñiga-García[b], Alberto Sánchez-González[b], Enrique Lozano-Álvarez[a,]*

[a]Unidad Académica de Sistemas Arrecifales, Instituto de Ciencias del Mar y Limnología, Universidad Nacional Autónoma de México, Puerto Morelos, Quintana Roo, México.
[b]Centro Interdisciplinario de Ciencias Marinas, Instituto Politécnico Nacional, La Paz, Baja California Sur, México.
[c]*Current address*: CONACYT–Instituto de Ciencias Marinas y Pesquerías, Universidad Veracruzana, Boca del Río, Veracruz, México.

*Corresponding author. Email: elozano@cmarl.unam.mx

Supplementary information

Supplementary tables

Supplementary Table S1. Summary of stable isotope information by reef. Mean ± SD $\delta^{15}N$ and $\delta^{13}C$ values (in parts per mil) of basal carbon sources and 48 species of consumers (invertebrates and fishes) on Limones and Bonanza reefs. Trophic position (TP) estimated for consumers according to Hussey et al.[1].

Sources/species	Trophic category	Reference	Limones Reef				Bonanza Reef			
			n	$\delta^{15}N$ ± SD	$\delta^{13}C$ ± SD	TP	n	$\delta^{15}N$ ± SD	$\delta^{13}C$ ± SD	TP
**Basal carbon sources**										
Particulate organic matter (POM)			3	2.85 ± 0.17	-18.39 ± 0.49		3	2.80 ± 0.56	-13.12 ± 1.89	
Algal turf			9	1.40 ± 0.87	-15.83 ± 2.76		10	1.78 ± 0.65	-18.76 ± 3.01	
Epiphytes			5	2.09 ± 0.51	-14.62 ± 1.27		5	2.19 ± 0.19	-11.97 ± 1.08	
Amphiroa tribulus			5	2.57 ± 0.19	-16.43 ± 0.51		5	0.50 ± 0.35	-8.78 ± 0.58	
Caulerpa racemosa			5	1.01 ± 0.11	-18.37 ± 0.30		5	0.96 ± 0.26	-18.35 ± 1.30	
Dictyota cervicornis			5	0.56 ± 0.22	-16.64 ± 0.76		5	0.95 ± 0.12	-15.22 ± 0.32	
Halimeda tuna			5	1.32 ± 0.08	-18.74 ± 0.77		5	0.52 ± 0.16	-18.62 ± 0.71	
**Consumers**										
Invertebrates										
Barbatia domingensis (B)	Ov	2	5	3.72 ± 0.22	-16.15 ± 0.07	1.60	5	3.63 ± 0.20	-14.53 ± 0.10	1.66
Calcinus tibicen (C)	Ov	3, 15	5	4.30 ± 0.50	-14.89 ± 1.43	1.77	5	3.39 ± 0.20	-15.00 ± 0.76	1.59
Caribachlamys ornata (B)	Ov	2	5	3.48 ± 0.28	-14.56 ± 0.33	1.53	5	4.23 ± 0.29	-13.59 ± 0.24	1.84
Cerithium litteratum (G)	Hv	2	5	4.11 ± 0.20	-14.87 ± 0.56	1.72	5	4.37 ± 0.28	-15.73 ± 0.45	1.88
Columbella mercatoria (G)	Hv	2	5	3.77 ± 0.16	-15.36 ± 0.87	1.61	5	3.98 ± 0.22	-15.01 ± 1.80	1.77
Coralliophila erosa (G)	Ov	2	5	5.06 ± 0.72	-10.47 ± 0.54	1.99	5	3.85 ± 0.40	-12.32 ± 0.87	1.73
Ctenoides mitis (B)	Ov	2	5	4.35 ± 0.81	-14.27 ± 0.47	1.79	5	4.91 ± 0.63	-13.22 ± 0.83	2.04
Cyphoma gibbosum (G)	Cv	2	5	5.98 ± 0.19	-13.54 ± 0.47	2.26	5	6.69 ± 0.41	-13.67 ± 0.36	2.56

Diadema antillarum (U)	Hv	4	5	5.37 ± 0.41	-13.51 ± 0.59	2.08	5	5.68 ± 0.64	-13.04 ± 1.39	2.26
Echinometra viridis (U)	Hv	5	5	5.35 ± 0.31	-15.01 ± 0.42	2.08	5	5.76 ± 0.61	-15.57 ± 0.38	2.29
Echinometra lucunter (U)	Hv	6	5	4.71 ± 0.47	-14.12 ± 0.19	1.89	5	5.13 ± 0.27	-15.05 ± 0.81	2.10
Eucidaris tribuloides (U)	Cv	6	5	8.50 ± 0.34	-13.63 ± 0.10	3.01	5	8.91 ± 0.56	-14.27 ± 0.65	3.22
Eupolymnia sp. (A)	Ov	7	5	3.57 ± 0.43	-15.87 ± 1.09	1.55	5	2.83 ± 0.72	-15.76 ± 0.90	1.43
Hemitoma octoradiata (G)	Hv	2	4	4.09 ± 0.20	-15.28 ± 1.21	1.71	5	3.94 ± 0.26	-12.62 ± 1.66	1.75
Holothuria floridana (H)	Ov	8	5	4.30 ± 0.54	-13.56 ± 0.51	1.77	4	5.00 ± 0.85	-13.93 ± 0.57	2.07
Lima caribaea (B)	Ov	2	5	4.67 ± 0.46	-15.02 ± 0.41	1.88	5	4.76 ± 0.29	-13.87 ± 0.52	1.99
Lithopoma caelatum (G)	Hv	2	5	3.43 ± 0.42	-14.75 ± 0.92	1.52	5	3.53 ± 0.31	-16.07 ± 0.43	1.63
Lithopoma tectum (G)	Hv	2	5	3.41 ± 0.38	-13.88 ± 0.83	1.51	5	3.76 ± 0.47	-16.82 ± 0.52	1.70
Mithraculus coryphe (C)	Hv	9	5	4.40 ± 0.22	-13.60 ± 0.42	1.80	5	5.33 ± 0.38	-11.90 ± 1.01	2.16
Mithrax aculeatus (C)	Ov	17	5	5.91 ± 0.32	-14.45 ± 0.41	2.24	5	5.66 ± 0.48	-15.60 ± 0.18	2.26
Morula nodulosa (G)	Cv	2	5	7.98 ± 0.33	-13.25 ± 0.41	2.85	5	7.58 ± 0.35	-13.48 ± 0.27	2.82
Octopus briareus (Ce)	Cv	10	4	6.46 ± 0.36	-12.81 ± 0.90	2.41	5	5.69 ± 0.63	-13.42 ± 1.06	2.27
Ophiocoma echinata (O)	Cv	11	5	6.87 ± 0.28	-13.19 ± 0.56	2.53	5	6.48 ± 0.26	-15.28 ± 0.62	2.50
Ophiocoma wendtii (O)	Cv	11	5	7.48 ± 0.51	-13.68 ± 0.52	2.70	5	7.48 ± 0.66	-15.24 ± 0.26	2.79
Paguristes anomalus (E)	Ov	12	5	3.65 ± 0.26	-14.60 ± 0.43	1.58	5	3.85 ± 0.15	-14.93 ± 0.56	1.73
Paguristes puncticeps (E)	Ov	12	5	3.73 ± 0.21	-14.17 ± 0.68	1.60	5	3.68 ± 0.24	-14.98 ± 0.95	1.68
Paguristes tortugae (E)	Ov	12	4	3.42 ± 0.54	-15.71 ± 0.84	1.51	5	4.71 ± 0.36	-14.63 ± 0.20	1.98
Pagurus brevidactylus (E)	Ov	12	5	3.05 ± 0.51	-13.55 ± 0.72	1.40	4	3.36 ± 0.45	-13.65 ± 0.72	1.58
Panulirus argus (L)	Ov	13	5	5.30 ± 0.55	-12.05 ± 0.90	2.22	5	5.59 ± 0.42	-12.70 ± 1.10	2.33
Panulirus guttatus (L)	Ov	13	5	6.83 ± 0.27	-12.42 ± 0.41	2.51	5	6.97 ± 0.16	-13.15 ± 0.49	2.64
Teleophrys ruber (C)	Hv	9	5	4.83 ± 0.41	-13.90 ± 0.40	1.93	4	4.59 ± 0.22	-14.48 ± 0.69	1.95
Stenoplax purpurascens (P)	Ov	2	4	3.99 ± 0.25	-14.04 ± 1.03	1.68	5	3.65 ± 0.36	-14.84 ± 1.56	1.67
Fish										
Acanthurus coeruleus	Hv	14, 16	3	6.17 ± 0.46	-15.50 ± 0.35	2.32	3	6.08 ± 0.20	-18.28 ± 0.77	2.38
Batrachoides gilberti	Cv	16	3	8.29 ± 0.07	-14.21 ± 0.63	2.94	5	7.82 ± 0.47	-13.66 ± 0.65	2.89
Cephalopholis cruentata	Cv	14, 16	5	9.12 ± 0.26	-13.42 ± 0.25	3.19	3	8.74 ± 1.04	-12.72 ± 0.74	3.16

Chaetodon capistratus	Cv	14, 16	3	7.66 ± 0.31	-12.46 ± 0.14	2.76	5	8.59 ± 0.35	-12.29 ± 0.47	3.12
Haemulon carbonarium	Cv	14, 16	5	9.23 ± 0.30	-13.11 ± 0.32	3.22	4	9.22 ± 0.25	-13.54 ± 0.28	3.31
Haemulon sciurus	Cv	14, 16	5	9.03 ± 0.56	-13.16 ± 1.02	3.16	5	8.17 ± 0.24	-13.01 ± 0.53	3.00
Lutjanus apodus	Cv	14, 16	3	9.41 ± 0.25	-12.77 ± 0.49	3.27	3	9.12 ± 1.18	-13.27 ± 0.33	3.28
Lutjanus griseus	Cv	14, 16	4	9.28 ± 0.60	-11.58 ± 1.52	3.24	5	9.14 ± 0.15	-13.08 ± 0.39	3.28
Ocyurus chrysurus	Ov	14, 16	3	7.62 ± 0.46	-13.50 ± 0.45	2.75	3	8.65 ± 0.17	-13.83 ± 0.09	3.14
Pempheris schomburgkii	Cv	14, 16	4	9.17 ± 0.16	-15.52 ± 0.49	3.20	5	9.14 ± 0.39	-14.33 ± 0.53	3.28
Pomacanthus paru	Ov	14, 16	3	7.40 ± 0.44	-18.39 ± 0.52	2.68	3	7.10 ± 0.78	-20.70 ± 0.87	2.68
Sargocentron vexillarium	Cv	14, 16	5	8.70 ± 0.12	-12.72 ± 0.71	3.06	4	8.80 ± 0.54	-13.52 ± 0.96	3.18
Scarus iseri	Hv	16	5	6.79 ± 0.21	-15.13 ± 0.99	2.50	3	5.51 ± 0.93	-14.71 ± 0.96	2.21
Sparisoma aurofrenatum	Hv	14, 16	3	6.14 ± 0.14	-16.31 ± 0.92	2.31	4	5.97 ± 0.39	-16.57 ± 0.51	2.35
Sparisoma viride	Hv	14, 16	3	5.47 ± 0.20	-14.83 ± 0.62	2.11	4	5.26 ± 0.36	-16.26 ± 0.42	2.14
Stegastes diencaeus	Ov	16	5	8.30 ± 0.31	-13.98 ± 0.92	2.95	5	8.12 ± 0.43	-14.91 ± 0.88	2.98

A: annelid, B: bivalve, C: crab, Ce: cephalopod, E: hermit crab, G: gastropod, H: holothurian, O: ophiurid, L: lobster, P: polyplacophora, U: urchin, Hv: Herbivore, Ov: Omnivore, Cv: Carnivore.

References

1. Hussey, N. E. *et al.* Rescaling the trophic structure of marine food webs. *Ecol. Lett.* **17**, 239–250 (2014).
2. Petuch, E. J. & Myers, R. F. *Molluscan Communities of the Florida Keys and Adjacent Areas: Their Ecology*. (CRC Press, 2014).
3. Hazlett, B.A. The behavorial ecology of hermit crabs. *Ann. Rev. Ecol. Syst.* **12**, 1–22 (1981).
4. Netchy, K., Hallock, P., Lunz, K. S. & Daly, K. L. Epibenthic mobile invertebrate diversity organized by coral habitat in Florida. *Mar. Biodiv.* **46**, 451–463 (2016).
5. Brown-Saracino, J., Peckol, P., Allen Curran, H. & Robbart, M. L. Spatial variation in sea urchins, fish predators, and bioerosion rates on coral reefs of Belize. *Coral Reefs* **26**, 71–78 (2007).
6. McClintock, J. B., Klinger, T. S. & Lawrence, J. M. Feeding preferences of echinoids for plant and animal food models. *Bull. Mar. Sci.* **32**, 365–369 (1982).
7. Grémare, A. & Amouroux, J. M. Feeding responses of the tentaculate deposit-feeder *Eupolymnia nebulosa* (Annelida: Polychaeta): influence of sexual maturity. *Mar. Biol.* **107**, 315–319 (1990).

8. Patrick, R. (2004) *Rivers of the United States, Volume I: Estuaries*. (Wiley, 1994).
9. Butler, M. J. & Mojica, A. M. Herbivory by the Caribbean king crab on coral patch reefs. *Mar. Biol.* **159**: 2697–2706 (2012).
10. Martinez, A. S., Mendes, L. F, & Leite, T. S. Spatial distribution of epibenthic molluscs on a sandstone reef in the Northeast of Brazil. *Braz. J. Biol.* **72**, 287-298 (2012).
11. Fatemi, S. R. Diversity of Ophiuroidea from Lengeh Portand Qeshm Island in the Persian Gulf. *J. Fish. Aquat. Sci.* **5**, 42–48 (2010).
12. Benvenuto, C., Sartonio, G. & Gherardi, F. Foraging behaviour of the hermit crab *Clibanarius erythropus* in a Mediterranean shore. *J. Mar. Biol. Assoc. U. K.* **83**, 457–461 (2003).
13. Briones-Fourzán, P., Castañeda-Fernández de Lara, V., Lozano-Álvarez, E. & Estrada-Olivo, J. Feeding ecology of the three juvenile phases of the spiny lobster *Panulirus argus* in a tropical reef lagoon. *Mar. Biol.* **142**, 855–865 (2003).
14. Randall, J. E. Food habits of reef fishes of the West Indies. *Stud. Trop. Oceanogr*. **5**, 655–847 (1967).
15. Poelen, J. H., Simons, J. D., & Mungall, C. J. Global biotic interactions: an open infrastructure to share and analyze species-interaction datasets. *Ecol. Inform.* **24**, 148–159 (2014).
16. Froese, R. & Pauly, D. (eds.) FishBase. World Wide Web electronic publication. www.fishbase.org, version (06/2017).
17. Winfree, R. A. & Weinstein, S. Food habits of the Caribbean king crab *Mithrax spinosissimus* (Lamarck). *Proc. Gulf Carib. Fish. Inst.* **39**, 458–464 (1989).

Supplementary Table S2. Stable isotopes comparisons between reefs. Results of Student's *t* test of the null hypothesis of no significant differences in the mean $\delta^{15}N$ and $\delta^{13}C$ of basal carbon sources and consumers between Limones and Bonanza reefs.

	$\delta^{15}N$			$\delta^{13}C$		
	t	**df**	**p**	*t*	**df**	**p**
**Basal carbon sources**						
POM	0.14	4	ns	-4.68	4	**
Algal turf	-1.08	17	ns	2.21	17	*
Epiphytes	-0.40	8	ns	-3.54	8	*
Amphiroa tribulus	11.72	8	***	-22.03	8	***
Caulerpa racemosa	0.41	8	ns	-0.03	8	ns
Dictyota cervicornis	-3.59	8	**	-3.88	8	**
Halimeda tuna	9.63	7	***	-0.26	8	ns
**Consumers**						
Invertebrates						
Barbatia domingensis	0.72	8	ns	-29.40	8	***
Calcinus tibicen	3.80	8	**	0.15	8	ns
Caribachlamys ornata	-4.15	8	**	-5.40	8	***
Cerithium litteratum	-1.70	8	ns	2.71	8	*
Columbella mercatoria	-1.77	8	ns	-0.39	8	ns
Coralliophila erosa	5.28	8	***	4.04	8	**
Ctenoides mitis	-1.21	8	ns	-2.49	8	*
Cyphoma gibbosum	-3.22	7	*	0.47	7	ns
Diadema antillarum	-0.92	8	ns	-0.70	8	ns
Echinometra viridis	-1.34	8	ns	2.21	8	ns
Eucidaris lucunter	-1.73	8	ns	2.49	8	*
Eucidaris tribuloides	-1.40	8	ns	2.19	8	ns
Eupolymnia sp.	1.97	8	ns	-0.18	8	ns
Hemitoma octoradiata	1.39	7	ns	-3.60	7	**
Holothuria floridana	-1.53	7	ns	1.02	7	ns
Lima caribaea	-0.39	8	ns	-3.86	8	**
Lithopoma caelatum	-0.40	8	ns	2.96	8	*
Lithopoma tectum	-1.28	8	ns	6.74	8	***
Mithraculus coryphe	-4.70	8	**	-3.45	8	**
Mithraculus ruber	1.04	7	ns	1.60	7	ns
Mithrax aculeatus	0.96	8	ns	5.72	8	***
Morula nodulosa	1.83	8	ns	1.03	8	ns
Octopus briareus	2.17	7	ns	0.90	7	ns
Ophiocoma echinata	2.29	8	ns	5.58	8	***
Ophiocoma wendtii	-0.01	8	ns	6.03	8	***
Paguristes anomalus	-1.47	8	ns	1.06	8	ns
Paguristes puncticeps	0.29	8	ns	1.55	8	ns

Paguristes tortugae	-4.36	7	**	-2.83	7	*
Pagurus brevidactylus	-0.97	7	ns	0.20	7	ns
Panulirus argus	-0.92	8	ns	0.64	8	ns
Panulirus guttatus	-1.03	8	ns	2.58	8	*
Stenoplax purpurascens	1.49	7	ns	1.52	7	ns
Fishes						
Acanthurus coeruleus	0.02	6	ns	1.71	6	ns
Batrachoides gilberti	2.72	2	ns	-1.03	2	ns
Cephalopholis cruentata	0.82	6	ns	-2.05	6	ns
Chaetodon capistratus	-3.51	4	*	-0.90	4	ns
Haemulon carbonarium	0.78	8	ns	1.09	8	ns
Haemulon sciurus	2.50	9	*	-0.56	9	ns
Lutjanus apodus	0.42	4	ns	1.49	4	ns
Lutjanus griseus	0.55	6	ns	1.76	6	ns
Ocyurus chrysurus	-4.01	5	*	1.26	5	ns
Pempheris schomburgkii	-0.39	6	ns	-4.29	6	**
Pomacanthus paru	-0.22	5	ns	-0.14	5	ns
Sargocentron vexillarium	-0.43	7	ns	1.45	7	ns
Scarus iseri	1.38	6	ns	0.02	6	ns
Sparisoma aurofrenatum	1.08	4	ns	0.66	4	ns
Sparisoma viride	-0.18	7	ns	3.38	7	*
Stegastes diencaeus	1.16	9	ns	1.50	9	ns

n.s. = not significant ($p > 0.05$); *$p < 0.05$; **$p < 0.01$; ***$p < 0.001$
